# Therapeutic intervention with sinomenine and irisin to preserve cardiac function after ischaemia–reperfusion injury

**DOI:** 10.1113/EP093586

**Published:** 2026-07-14

**Authors:** Lianjun Wu, Sining Bi, Amany Belal, Zhuzhu Liu

**Affiliations:** ^1^ Department of Cardiology Harbin 242 Hospital Harbin China; ^2^ Department of Cardiology Jinan Third People's Hospital Jinan China; ^3^ College of Pharmacy, Pharmaceutical Chemistry Department Taif University Taif Saudi Arabia; ^4^ Department of Cardiovascular Medicine Xi'an International Medical Center Hospital Xi'an China

**Keywords:** Irisin, mitophagy, myocardial ischaemia–reperfusion injury, pyroptosis, sinomenine

## Abstract

Despite advancements in reperfusion therapy, myocardial ischaemia–reperfusion (IR) injury remains a major clinical challenge. This study investigated whether a novel dual‐target pre‐conditioning strategy using sinomenine and irisin could enhance myocardial resistance against IR injury. Ninety male Sprague–Dawley rats were utilized. Protocol 1 evaluated cardioprotection by assigning rats to Sham, IR, sinomenine, irisin or a combination of both agents, with pharmacological pre‐treatments administered for 7 days prior to surgery. To model IR injury in vivo, animals underwent 30 min surgical ligation of the left anterior descending coronary artery followed by 24 h reperfusion. Combined pre‐treatment exerted superior protection, significantly reducing infarct size (*P* = 0.0187) and serum cardiotroponin‐I (*P* = 0.0028), while preserving myocardial architecture. Echocardiography and haemodynamic monitoring confirmed significantly enhanced ejection fraction (*P *< 0.0001), fractional shortening (*P* = 0.0002), developed pressure (*P* = 0.0001), +d*P*/d*t* (*P *< 0.0001), and −d*P*/d*t* (*P* = 0.0003), alongside reduced left ventricular internal diameter at end‐systole (*P *< 0.0001) and end‐diastole (*P* = 0.0004), as well as decreased end‐diastolic pressure (*P* = 0.0128) in the combination group. Protocol 2 investigated mechanisms using mitochondrial division inhibitor 1 (Mdivi‐1). Combined pre‐treatment mitigated oxidative stress, suppressed pro‐inflammatory cytokines and inhibited the nucleotide‐binding oligomerization domain‐like receptor protein 3 (NLRP3) inflammasome, evidenced by significant reductions in NLRP3, apoptosis‐associated speck‐like protein containing a CARD, and cleaved Gasdermin D. Conversely, it significantly upregulated PTEN‐induced putative kinase 1 (PINK1) and Parkin. Notably, Mdivi‐1 abrogated these benefits, confirming that the enhanced protection is mediated through the activation of the PINK1/Parkin‐dependent mitophagy pathway. These results suggest that pharmacological pre‐conditioning with sinomenine and irisin offers a potent strategy against IR injury by modulating the mitophagy–pyroptosis axis.

## Abstract (highlights)

- **What is the central question of this study?**
  Does combined pre‐treatment with sinomenine and irisin provide superior cardioprotection against myocardial ischaemia–reperfusion injury, and is this effect mediated through suppression of pyroptosis and activation of PINK1/Parkin‐dependent mitophagy?
- **What is the main finding and its importance?**
  The dual pre‐treatment markedly reduced infarct size, improved cardiac function, suppressed NLRP3‐driven pyroptosis, and enhanced mitophagy. Pharmacological inhibition of mitophagy largely abrogated these protective effects, indicating that activation of mitophagy is a key mechanistic mediator of the observed cardioprotection. These findings support a multi‐target strategy integrating mitochondrial quality control and inflammatory cell death modulation, with promising translational potential beyond single‐agent approaches.

## 1 INTRODUCTION

Myocardial ischaemia–reperfusion (IR) injury represents a serious pathological process characterized as the paradoxical intensification of heart tissue damage during the reestablishment of blood flow following ischaemia (He et al., 2022). Ischaemia causes severe metabolic derangement due to the consumption of intracellular concentrations of ATP and intracellular acidosis with ionic imbalance and calcium overload of cardiomyocytes. Moreover, reperfusion significantly exacerbates tissue damage through the rapid generation of reactive oxygen species (ROS), oxidative stress, opening of the mitochondrial permeability transition pore (mPTP), and activation of multiple programmed cell death pathways, including apoptosis, pyroptosis and necroptosis (Buja, 2023). Furthermore, inflammation triggered by robust ROS‐induced oxidative stress significantly exacerbates heart tissue damage and contributes to microvascular dysfunction (Dhalla et al., 2022). Taken together, these interrelated processes indicate that myocardial IR injury is driven by multifactorial and convergent signalling pathways. Thus, an effective therapeutic strategy should target multiple pathways to reduce cardiac tissue injury and improve overall cardiac function (Sagris et al., 2024).

Pyroptosis is a distinct form of programmed cell death, characterized by inflammatory rupture of the cell membrane, and is increasingly recognized as a pivotal contributor to myocardial IR injury (Liu et al., 2022). This process is primarily mediated by activation of the nucleotide‐binding oligomerization domain‐like receptor protein 3 (NLRP3) inflammasome, which consists of NLRP3, the adaptor apoptosis‐associated speck‐like protein containing a CARD (ASC), and pro‐caspase‐1. Upon activation, caspase‐1 is recruited and cleaves Gasdermin D (GSDMD), leading to the formation of membrane pores, the release of pro‐inflammatory cytokines such as interleukin (IL)‐18 and IL‐1β, and subsequent inflammatory cell death. The resulting cytokine storm amplifies the inflammatory response, exacerbates cardiomyocyte injury and impairs myocardial function (Ye et al., 2022). Notably, accumulating dysfunctional mitochondria and excessive mitochondrial ROS generation during IR act as key upstream triggers of NLRP3 activation, linking mitochondrial injury directly to pyroptotic signalling. In this context, impaired mitophagy permits the persistence of damaged mitochondria, thereby enhancing inflammasome activation, whereas efficient PTEN‐induced putative kinase 1 (PINK1)/Parkin‐mediated mitophagy limits mitochondrial ROS production and mitochondrial DNA release, restrains NLRP3 activation and attenuates pyroptosis (Wu et al., 2022). This tight interplay between mitochondrial quality control and inflammatory cell death underscores the mechanistic crosstalk driving myocardial IR injury and highlights the therapeutic value of simultaneously modulating both pathways (Toldo et al., 2018; Xu et al., 2024).

Despite extensive experimental investigation, effective pharmacological interventions for myocardial IR injury remain elusive, primarily because its pathogenesis is multifactorial and involves tightly interconnected signalling cascades that cannot be sufficiently modulated by single‐target approaches. Accordingly, most monotherapies yield only partial cardioprotection, as they influence a limited component of a highly integrated pathological network (Hausenloy & Heusch, 2019). The dynamic interplay among oxidative stress, inflammation, mitochondrial dysfunction and apoptosis further constrains the capacity of monotherapies to confer robust cardioprotection (Andreadou et al., 2018). Hence, the strategy of combination therapy has been identified as the more effective treatment for the simultaneous targeting of multiple molecular pathways involved in myocardial IR injury (Mokhtari et al., 2023). Sinomenine has been identified as the biologically active chemical present in the plant *Sinomenium acutum*. This chemical has potent antioxidant, anti‐inflammatory and immunosuppressive properties (Lu et al., 2022). Mechanistically, it suppresses nuclear factor κ‐light‐chain‐enhancer of activated B cells (NF‐κB) signalling and inhibits activation of the NLRP3 inflammasome, thereby attenuating pyroptosis, reducing oxidative stress and limiting inflammation within the ischaemic myocardium (Xia et al., 2022; Zhang et al., 2021). In parallel with these anti‐inflammatory properties, targeting mitochondrial homeostasis represents another critical therapeutic axis in myocardial IR injury. Irisin has been identified as the chemical released during exercise as a product of the enzymatic breakdown of the protein kinases adiponectin receptor (ADIPOR) 1 and ADIPOR2 present in the blood. It has been recognized as the link between physical exercise and the prevention of the development of insulin resistance (Lu et al., 2024). Studies have shown that irisin can enhance mitochondrial quality by promoting mitophagy, while simultaneously mitigating apoptosis and inflammatory responses (Jin & Piao, 2024; Xin et al., 2020). Taken together, the mechanistic complementarity of sinomenine in suppressing inflammation‐driven pyroptotic signalling and irisin in preserving mitochondrial function provides a strong biological rationale for their combined administration as a multitarget strategy to achieve superior cardioprotection in myocardial IR injury.

Based on the aforementioned pharmacological profiles, the concurrent administration of sinomenine and irisin offers a promising multi‐targeted approach to address the complex pathophysiology of myocardial IR injury. Consequently, this study investigated the potential combined cardioprotective effects of these two agents in a rat model of myocardial IR injury. Special emphasis was placed on modulating the key pathological signalling pathways and oxidative stress markers that underlie IR‐induced damage. This approach is innovative, as it targets multiple synchronized processes that are often not adequately managed by monotherapy. These findings are of critical importance for bridging the translational gap from preclinical models to clinical applications in pursuit of novel multi‐modal therapeutic strategies for ischaemic heart disease.

## 2 METHODS

### 2.1 Ethical approval

All experimental procedures were carried out in accordance with the ethical guidelines approved by the Zhinanzhen Biology Ethics Committee (Approval Reference Number: A2024000922) and complied with the animal experiment policies of *Experimental Physiology*. This study adhered to the Animals in Research: Reporting In Vivo Experiments (ARRIVE) 2.0 guidelines and the National Institutes of Health (8th ed., rev. 2011) standards. For surgical procedures, animals were anaesthetized with a combination of ketamine (60 mg/kg) and xylazine (10 mg/kg) administered via intraperitoneal injection. At the end of the study, in accordance with the American Veterinary Medical Association (AVMA) Guidelines, animals were humanely euthanized under deep anaesthesia (induced by a repeat dose of ketamine/xylazine, 60/10 mg/kg, intraperitoneally), followed by thoracotomy and heart excision, leading to exsanguination and permanent cessation of circulatory function. Cardiac tissues were then harvested for downstream analyses, including infarct size measurement, histopathology and molecular assessments.

### 2.2 Experimental animals

Ninety male Sprague–Dawley rats, aged 8–10 weeks, weighing 200–250 g, were obtained from the accredited laboratory animal breeding facility of Xi'an International Medical Center Hospital. The animals were maintained under standard laboratory conditions: temperature 20–24°C, relative humidity 55%, and a light–dark cycle of 12 h each to simulate natural living conditions. During the study, the rats had unlimited access to standard rodent food and fresh water. The animals were acclimatized to the laboratory environment for at least 1 week before the start of the experiments to minimize stress and physiological variability.

### 2.3 Study design

The sample size for each group was determined by an a priori power analysis using G*Power software (v.3.1.9.7). Based on an α‐level of 0.05, a power of 0.80 and an expected effect size of 0.5 for the primary endpoint (infarct size), a minimum of six rats per subgroup were required.

Two distinct experimental protocols were randomly assigned to the rats to evaluate different facets of cardioprotection. Protocol 1 (*n* = 60) aimed to evaluate the effectiveness of combined pre‐treatment compared to individual pre‐treatments. Rats were randomly divided into five groups (*n* = 12 per group):

1. Sham group: Rats underwent open‐heart surgery without myocardial IR injury.
2. IR group: Rats subjected to 30 min of ischaemia followed by 24 h of reperfusion.
3. IR + Sin group: Rats received intraperitoneal injections of sinomenine (purity ≥98%; Sigma‐Aldrich, St Louis, MO, USA) at a dose of 80 mg/kg/day for seven consecutive days prior to surgery (Xia et al., 2022). The sinomenine powder was dissolved in 0.9% normal saline as the vehicle.
4. IR + Iri group: Rats received recombinant rat irisin (0.5 mg/kg/day; Cloud‐Clone Corp., Wuhan, China) via intraperitoneal injection for 7 days prior to surgery (Xu et al., 2022). The lyophilized irisin was reconstituted in sterile 0.9% normal saline according to the manufacturer's instructions.
5. IR + Sin + Iri group: Rats received both sinomenine and irisin according to the aforementioned dosages and schedules.

To ensure standardized conditions, rats in the Sham and IR groups received an equivalent volume of sterile 0.9% normal saline as a vehicle following the same administration schedule as the pre‐treatment groups. Twenty‐four hours after the surgery, each group was divided into two analytical subsets (*n* = 6 each). The first subset was anaesthetized (ketamine 60 mg/kg and xylazine 10 mg/kg, intraperitoneally) for cardiac function evaluation via echocardiography and haemodynamic measurements. Subsequently, blood was collected via cardiac puncture for serum cardiotroponin‐I (cTn‐I) quantification. These animals were then euthanized by thoracotomy and heart excision for histopathological analysis. In the second subset (*n* = 6), myocardial infarct size was measured following euthanasia.

Protocol 2 (*n* = 30) investigated the molecular mechanisms underlying the enhanced cardioprotection, specifically focusing on pyroptosis and mitophagy pathways. To investigate the mechanistic involvement of mitophagy, the mitochondrial division inhibitor 1 (Mdivi‐1) was administered. Rats were divided into five groups (*n* = 6 per group):

1. Sham group: As described in Protocol 1.
2. IR group: As described in Protocol 1.
3. IR + Sin + Iri group: As described in Protocol 1.
4. IR + Mdivi‐1 group: Rats were pre‐treated with Mdivi‐1 (MedChemExpress, Monmouth Junction, NJ, USA) at a dose of 1 mg/kg/day via intraperitoneal injection for seven consecutive days prior to surgery to pharmacologically inhibit mitophagy, as previously described (Xu & Yu, 2024). The Mdivi‐1 was initially dissolved in dimethyl sulfoxide (DMSO) and subsequently diluted in sterile 0.9% normal saline (final DMSO concentration <1%).
5. IR + Sin + Iri + Mdivi‐1 group: Rats received Mdivi‐1 in combination with sinomenine and irisin according to the aforementioned dosages and schedules.

To ensure a rigorous control, rats in the Sham and IR groups received an equivalent volume of the vehicle (sterile 0.9% normal saline containing <1% DMSO) via intraperitoneal injection according to the same 7‐day schedule. One day post‐surgery, rats were anaesthetized and euthanized by heart excision. Cardiac tissues were harvested for molecular and biochemical assessments, including western blotting for pyroptosis and mitophagy‐related proteins, inflammatory cytokine quantification and oxidative stress measurements.

### 2.4 Myocardial IR injury model

To induce myocardial IR injury, rats assigned to the IR groups were anaesthetized via intraperitoneal injection of ketamine (60 mg/kg) and xylazine (10 mg/kg). During the entire surgical procedure, animals were continuously monitored for electrocardiographic (ECG) activity, respiratory rate and overall perfusion. Following endotracheal intubation, animals were mechanically ventilated using a small animal ventilator (Taimeng Technology, Chengdu, China) set to deliver 80–90 breaths per minute. A left thoracotomy was performed through a midline incision along the left border of the sternum to expose the heart. The left anterior descending (LAD) coronary artery was identified and ligated with a suture. The induction of ischaemia was confirmed by whitening of the left ventricular apex and elevation of the ST segment on the ECG. After 30 min of ischaemia, the ligature was removed to initiate reperfusion, which was confirmed by the restoration of colour in the anterior left ventricular wall and normalization of the ST segment, and reperfusion was maintained for 24 h. Throughout the recovery period, animals were closely monitored for heart rate (HR), respiratory function, mobility and overall condition. Analgesia was provided with buprenorphine (0.05 mg/kg, subcutaneously, every 12 h) according to institutional guidelines, along with supportive care, to minimize pain and distress. Sham‐operated controls underwent the same procedure, except that the LAD artery was threaded but not ligated (Xu et al., 2014).

### 2.5 Cardiac function assessment

#### 2.5.1 Echocardiographic evaluation

Rats were anaesthetized and placed supine on a heated platform in order to maintain body temperature. Depilation of the chest area with a hair removal cream improved ultrasound signal transmission. A high‐frequency ultrasound probe (∼30 MHz) was connected with a small animal echocardiography system (Esaote VET, Esaote SpA, Genoa, Italy) for acquiring two‐dimensional and M‐mode images of the heart. Standard parasternal long‐axis and short‐axis views were used to measure cardiac parameters including left ventricular ejection fraction (EF), fractional shortening (FS), and left ventricular internal diameter at end‐systole (LVIDs) and end‐diastole (LVIDd). HR and rhythm were monitored continuously throughout image acquisition (Mokhtari & Badalzadeh, 2023).

#### 2.5.2 Haemodynamic evaluation

Cardiac haemodynamics were evaluated invasively using a microtip pressure catheter connected to a PowerLab data acquisition system (ADInstruments, Sydney, Australia). Under anaesthesia, the right carotid artery was carefully dissected, and a PE‐50 catheter was inserted and threaded into the ascending aorta to continuously monitor arterial pressure. The mean arterial pressure (MAP) and HR were continuously monitored and recorded for 20 min to establish baseline systemic haemodynamics. After the first 20‐min recording, the catheter was gently advanced retrogradely through the aortic valve into the left ventricle for direct ventricular pressure measurement. Parameters determined included left ventricular end‐diastolic pressure (LVEDP), left ventricular developed pressure (LVDP, calculated as the difference between left ventricular systolic pressure [LVSP] and LVEDP), and the maximum rates of pressure increase and decrease during ventricular contraction and relaxation (+d*P*/d*t*_max_ and −d*P*/d*t*_max_) (Mokhtari et al., 2023).

### 2.6 Measurement of serum cTn‐I levels

Serum cTn‐I levels were measured as a specific marker of myocardial injury. Blood was drawn by cardiac puncture, and the serum was separated by centrifugation at 1,000 *g* for 10 min. The serum was then aliquoted and stored at −80°C until analysis. Quantification of cTn‐I was performed using a rat‐validated ELISA kit (Life Diagnostics, West Chester, PA, USA) according to the manufacturer's instructions. All samples and standards were assayed in duplicate, and absorbance was measured at 450 nm using a microplate reader. Intra‐ and inter‐assay coefficients of variation were both below 10%.

### 2.7 Histopathological examination

Following haemodynamic measurements, rats were euthanized under deep anaesthesia. The left ventricular tissues were carefully excised, fixed in 4% paraformaldehyde for 24 h, and then processed for paraffin embedding. Serial sections of 5 µm thickness were prepared for staining. Tissue architecture was evaluated using haematoxylin and eosin (H&E) staining. Sections were examined systematically under a light microscope by a pathologist who was blinded to the experimental groups, and histopathological alterations – including myocyte degeneration, oedema, inflammatory cell infiltration and necrosis – were documented qualitatively.

### 2.8 Myocardial infarction measurement

Twenty‐four hours after reperfusion, the LAD artery was reoccluded, followed by infusion of 0.5% Evans Blue dye via the aortic cannula. The hearts were then removed and stored at −20°C. After 24 h, the left ventricles were transversely sectioned into five 2‐mm‐thick slices and incubated in 1% triphenyltetrazolium chloride (TTC) prepared in phosphate buffer (pH 7.4) at 37°C for 20 min. This staining procedure allowed differentiation of myocardial regions, where normal tissue appeared blue, ischaemic tissue stained light red, and infarcted tissue remained unstained, appearing pale white. Ventricular slices were photographed, and both TTC‐negative areas (infarcted regions) and TTC‐positive areas (area at risk) were assessed. Calculation of infarct size was expressed as a percentage of the area at risk and quantified using computerized planimetry with ImageJ software, version 1.6 (National Institutes of Health, Bethesda, MD, USA).

### 2.9 Western blot analysis

The expression levels of various proteins in myocardial tissue were measured by western blot analysis. Left ventricular tissue samples were collected and processed for analysis. Total proteins were extracted using radioimmunoprecipitation assay lysis buffer (Sigma‐Aldrich; R0278). After extraction, nucleoproteins were purified by centrifugation at 14,000 *g* for 15 min. The amount of protein was measured, and proteins were separated by sodium dodecyl sulfate‐polyacrylamide gel electrophoresis. Thereafter, proteins were transferred onto polyvinylidene difluoride membranes. Non‐specific binding was blocked with 5% skimmed milk. Membranes were incubated overnight with primary antibodies at 4°C, as follows: anti‐NLRP3 (1:200; Novus Biologicals, Littleton, CO, USA; Cat. No. NBP2‐12446), anti‐ASC (1:200; Santa Cruz Biotechnology, Dallas, TX, USA; sc‐514414), anti‐N‐terminal gasdermin D (GSDMD‐N; 1:200; Santa Cruz Biotechnology; sc‐393581), anti‐PINK1 (1:1000; Abcam, Cambridge, UK; ab216144), anti‐Parkin (1:1000; Abcam; ab15954), and anti‐β‐actin (1:1000; Santa Cruz Biotechnology; sc‐47778). After incubation with primary antibodies, membranes were incubated with horseradish peroxidase‐conjugated secondary antibodies (1:2000; Proteintech, Rosemont, IL, USA; SA00001‐1 and SA00001‐2) for 1 h at 37°C. Protein bands were visualized using an enhanced chemiluminescence detection system and quantified according to their signal intensities, normalized to the expression of β‐actin as a loading control.

### 2.10 Assessment of pro‐inflammatory cytokine levels and oxidative stress status

Left ventricular tissues were homogenized mechanically and centrifuged (1,400 *g*) for 10 min at 4°C. The clear supernatants obtained were assayed for the pro‐inflammatory cytokines IL‐18 and IL‐1β using commercial ELISA kits (R&D Systems, Minneapolis, MN, USA) according to the manufacturer's guidelines. Cytokine concentration was determined based on the absorbance measured at 450 nm. Oxidative stress in the left ventricular samples was assessed based on several biochemical assays. Lipid peroxidation was analysed by determination of the levels of malondialdehyde (MDA) with the thiobarbituric acid reactive substances (TBARS) method, which forms a pink chromogen detectable at 532 nm. Superoxide dismutase (SOD) activity was assayed using its ability to inhibit the reduction of nitroblue tetrazolium (NBT) by superoxide radicals at 560 nm. Glutathione peroxidase (GPx) activity was quantified in a coupled enzymatic assay in which NADPH consumption was monitored at 340 nm as an indicator of glutathione oxidation.

### 2.11 Statistical analysis

Data were analysed using GraphPad Prism software (version 9.5.1, GraphPad Software, Boston, MA, USA). Normality of data distribution was assessed using the Shapiro–Wilk test. Statistical analysis was conducted within a hypothesis‐driven framework with pre‐specified comparisons. The primary comparisons were defined as differences between each pre‐treatment group and the IR control group, while secondary analyses included inter‐treatment comparisons (e.g., monotherapy vs. combination therapy) and mechanistic comparisons involving inhibitor‐treated groups. For normally distributed data, one‐way analysis of variance (ANOVA) was performed followed by Tukey's *post hoc* test to account for multiple pairwise comparisons. Although Dunnett's test is suitable for comparisons versus a single control, Tukey's test was applied to accommodate both control‐based and inter‐group comparisons within the same analytical framework. Data are presented as means ± standard deviation (SD). Statistical significance was defined as *P* < 0.05.

## 3 RESULTS

### 3.1 Effects of sinomenine and irisin on infarct size, cardiac injury marker, and histopathology

**FIGURE 1:**
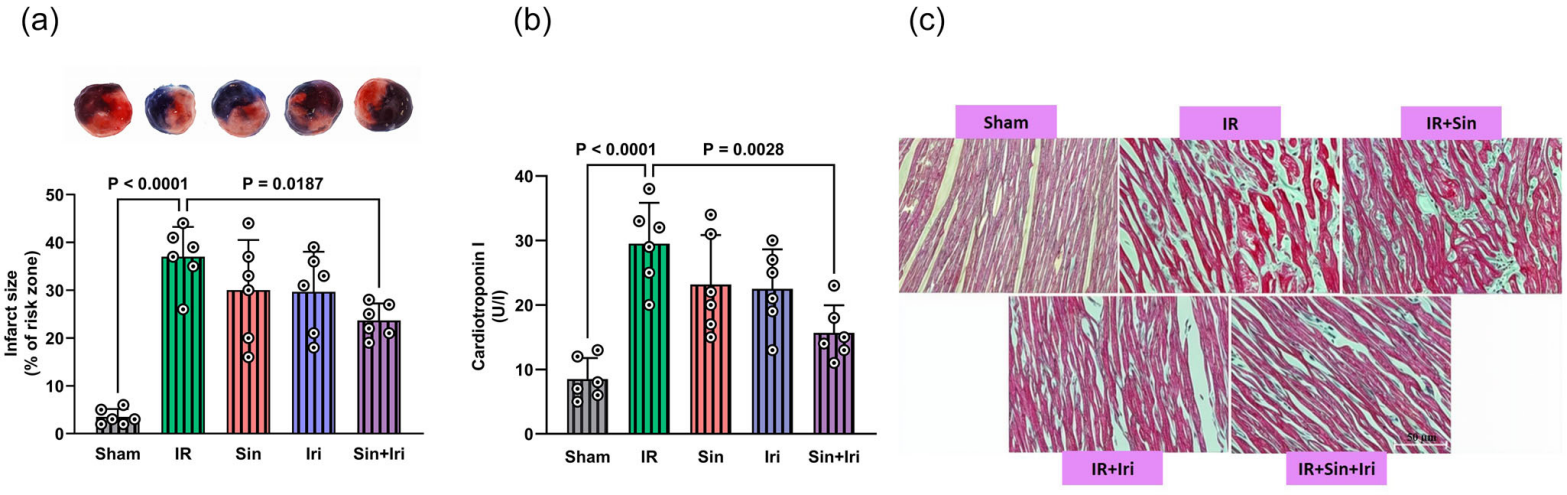
Infarct size of the myocardium, levels of cardiac injury biomarker and histopathological changes between experimental groups. (a) Infarct size, (b) serum cardiotroponin‐I concentrations, and (c) representative histopathological changes (original magnification ×40). Data are presented as means ± standard deviation (*n* = 6 per group) and analysed statistically by one‐way ANOVA with Tukey's *post hoc* test. IR, ischaemia–reperfusion; Iri, irisin; Sin, sinomenine.

Compared with the Sham group, myocardial infarct size was markedly increased in the IR group (37.00 ± 6.22% vs. 3.50 ± 1.64%, *P* < 0.0001), corresponding to a ∼33.50% absolute increase (Figure 1a). Compared with the IR group, pre‐treatment with either sinomenine (30.00 ± 10.49%) or irisin (29.67 ± 8.38%) did not significantly reduce infarct size (7.00% and 7.33% absolute reductions, respectively) (Figure 1a). In contrast, combined administration of sinomenine and irisin significantly reduced infarct size to 23.67 ± 3.55%, representing a 13.33% absolute reduction compared with the IR group (*P* = 0.0187) (Figure 1a). It is worth noting that the minor infarct size observed in the Sham group is attributable to baseline surgical stress associated with thoracotomy, pericardiotomy and suture placement around the LAD artery without ligation, and does not reflect ischaemic necrosis.

Serum cTn‐I levels were measured to assess myocardial injury across the experimental groups. Compared with the Sham group, myocardial IR markedly increased cTn‐I levels (29.50 ± 6.34 ng/mL vs. 8.50 ± 3.27 ng/mL, *P* < 0.0001), corresponding to a 21.00 ng/mL absolute increase (Figure 1b). Compared with the IR group, pre‐treatment with either sinomenine (23.17 ± 7.67 ng/mL) or irisin (22.50 ± 6.12 ng/mL) did not significantly reduce serum cTn‐I levels (6.33% and 7.00% absolute reductions, respectively) (Figure 1b). In contrast, combined administration of sinomenine and irisin significantly decreased cTn‐I levels to 15.67 ± 4.27 ng/mL, corresponding to a 13.83 ng/mL absolute reduction compared with the IR group (*P* = 0.0028) (Figure 1b). The Sham group showed minimal cTn‐I levels (8.50 ± 3.27 ng/mL), which are attributed to baseline surgical stress and not ischaemic injury.

Histopathological examination of cardiac tissues stained with H&E in the Sham group showed well‐organized cardiomyocytes arranged in parallel rows with a clear fibre architecture and uniform cytoplasm. There was no evidence of fibre damage, necrosis, lymphocyte infiltration or any inflammatory cells. The IR group, however, presented with necrosis, interstitial oedema, widened intercellular spaces, myofibrillar thinning and infiltration of inflammatory cells. Pre‐treatment with sinomenine or irisin alone partially improved these pathological changes, but could not completely prevent them. It was further observed that combined therapy significantly reversed all histopathological abnormalities toward normal morphology, as seen in the Sham group, thereby showing greater therapeutic efficacy than either pre‐treatment alone (Figure 1c).

### 3.2 Effects of sinomenine and irisin on echocardiographic parameters

**FIGURE 2:**
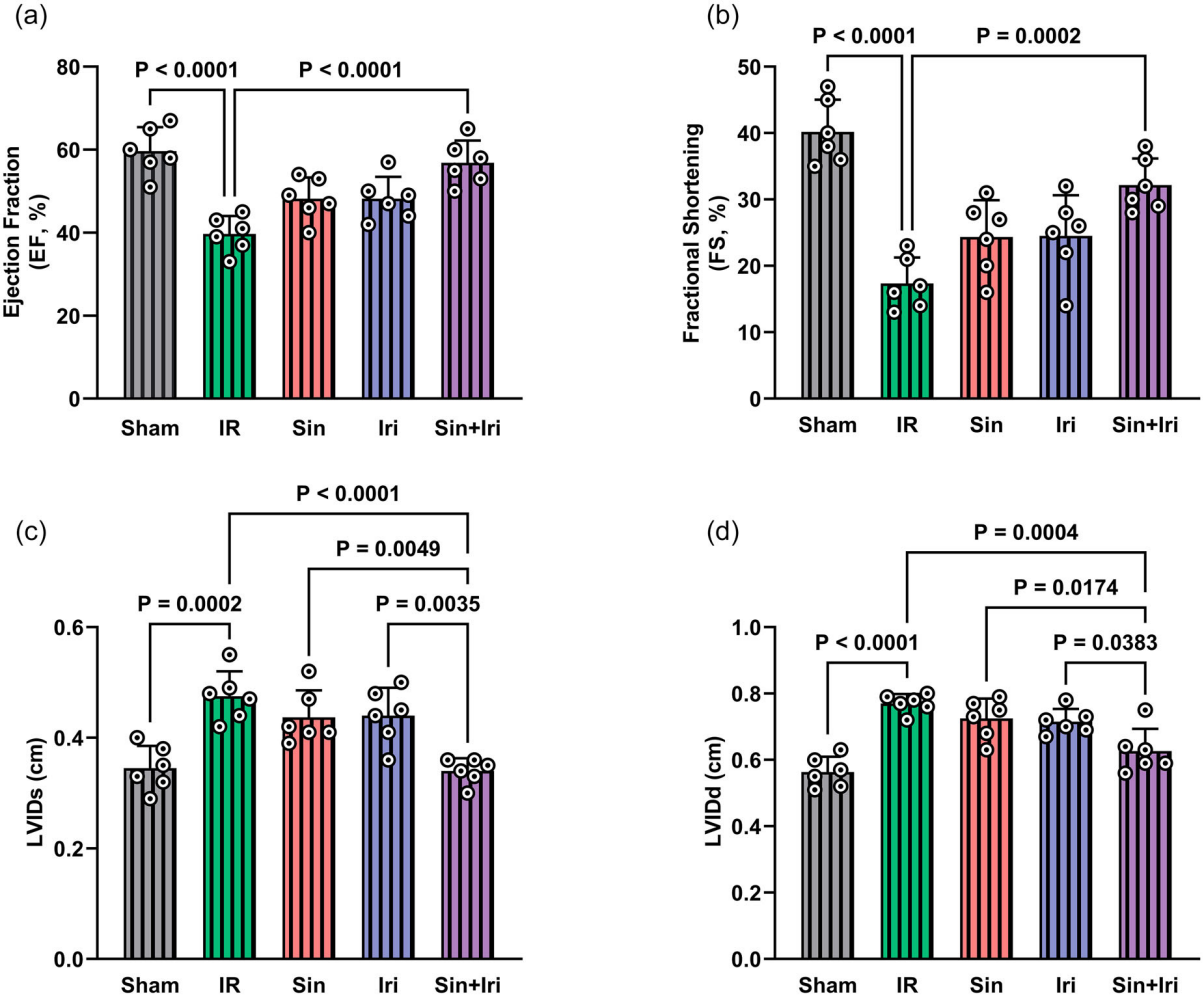
Results of a comprehensive echocardiographic evaluation of cardiac function across groups. Panels depict important parameters such as ejection fraction (EF) (a), fractional shortening (FS) (b), and left ventricular internal diameter at end‐systole (LVIDs) and end‐diastole (LVIDd) (c and d, respectively). Data are presented as means ± standard deviation (*n* = 6 per group) and analysed statistically by one‐way ANOVA with Tukey's *post hoc* test. IR, ischaemia–reperfusion; Iri, irisin; Sin, sinomenine.

Compared with the Sham group, EF was markedly reduced in the IR group (39.67 ± 4.32% vs. 59.67 ± 5.78%, *P* < 0.0001), corresponding to a 20.00% absolute decrease (Figure 2a). Compared with the IR group, pre‐treatment with either sinomenine (48.17 ± 5.11%) or irisin (48.17 ± 5.26%) partially increased EF (8.50% absolute increases for both); however, these improvements did not reach the level observed in the Sham group. In contrast, combined administration of sinomenine and irisin significantly increased EF to 56.83 ± 5.34%, representing a 17.16% absolute increase compared with the IR group (*P < 0.0001*) (Figure 2a). Similarly, FS was significantly decreased in the IR group compared with the Sham group (17.33 ± 3.93% vs. 40.17 ± 4.87%, *P* < 0.0001), corresponding to a 22.84% absolute reduction (Figure 2b). Pre‐treatment with sinomenine (24.33 ± 5.53%) or irisin (24.50 ± 6.12%) modestly, but not significantly, improved FS, corresponding to absolute increases of 7.00% and 7.17% versus the IR group, respectively. Notably, combined pre‐treatment markedly increased FS to 32.17 ± 4.02%, corresponding to a 14.84% absolute increase compared with the IR group (*P* = 0.0002) (Figure 2b).

Results of left ventricular dimensions are as follows: LVIDs was significantly increased in the IR group compared with the Sham group (0.475 ± 0.045 cm vs. 0.345 ± 0.040 cm, *P* = 0.0002), corresponding to a 0.130 cm absolute increase (Figure 2c). Sinomenine (0.436 ± 0.048 cm) and irisin (0.440 ± 0.050 cm) slightly, but not significantly, reduced LVIDs, corresponding to absolute reductions of 0.039 cm and 0.035 cm versus the IR group, respectively. In contrast, combined administration significantly decreased LVIDs to 0.340 ± 0.022 cm, representing a 0.135 cm absolute reduction compared with the IR group (*P* < 0.0001), restoring values close to the Sham level (Figure 2c). Likewise, LVIDd was markedly increased in the IR group compared with the Sham group (0.770 ± 0.028 cm vs. 0.563 ± 0.046 cm, *P* < 0.0001), corresponding to a 0.207 cm absolute increase (Figure 2d). Pre‐treatment with sinomenine (0.725 ± 0.059 cm) or irisin (0.715 ± 0.038 cm) resulted in modest reductions (0.045 cm and 0.055 cm absolute decreases versus the IR group, respectively), which were not statistically significant. Importantly, co‐administration of sinomenine and irisin significantly reduced LVIDd to 0.626 ± 0.067 cm, corresponding to a 0.144 cm absolute reduction compared with the IR group (*P* = 0.0004), indicating attenuation of ventricular dilation (Figure 2d). The combined therapy also demonstrated superior efficacy over monotherapies, significantly decreasing LVIDs (*P* = 0.0049 vs. sinomenine; *P* = 0.0035 vs. irisin) and LVIDd (*P* = 0.0174 vs. sinomenine; *P* = 0.0383 vs. irisin) (Figure 2c,d).

### 3.3 Effects of sinomenine and irisin on haemodynamic indices

**FIGURE 3:**
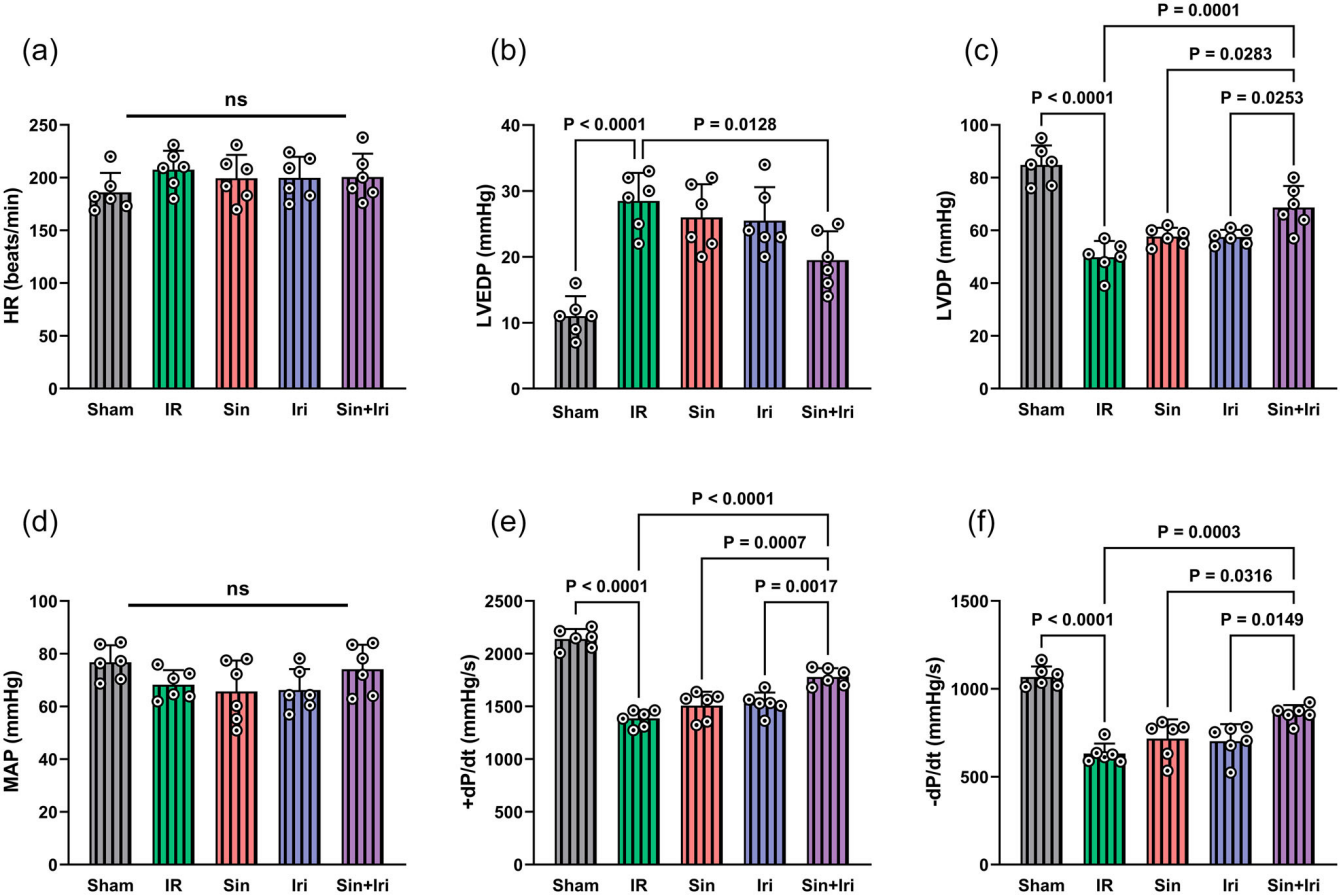
Haemodynamic parameters measured across experimental groups. Heart rate (HR) (a), left ventricular end‐diastolic pressure (LVEDP) (b), left ventricular developed pressure (LVDP) (c), mean arterial pressure (MAP) (d), and the maximum rates of pressure increase and decrease during ventricular contraction and relaxation (+d*P*/d*t*_max_ and −d*P*/d*t*_max_) (e and f, respectively). Data are expressed as means ± standard deviation (*n* = 6 per group) and statistically analysed using one‐way ANOVA with Tukey's *post hoc* test. IR, ischaemia–reperfusion; Iri, irisin; Sin, sinomenine.

No significant differences were observed among groups in HR or MAP (Figure 3a,d). Compared with the Sham group, IR markedly impaired haemodynamic function, as evidenced by a significant increase in LVEDP (28.50 ± 4.23 mmHg vs. 11.00 ± 3.03 mmHg, *P* < 0.0001) and significant reductions in LVDP (49.83 ± 6.17 mmHg vs. 84.83 ± 7.36 mmHg, *P* < 0.0001), +d*P*/d*t* (1386 ± 79.06 mmHg/s vs. 2139 ± 93.83 mmHg/s, *P* < 0.0001) and –d*P*/d*t* (631.50 ± 57.00 mmHg/s vs. 1068 ± 58.73 mmHg/s, *P* < 0.0001) (Figure 3b,c,e,f). Monotherapy with either sinomenine or irisin did not significantly affect these parameters compared with the IR group. In contrast, combined administration of sinomenine and irisin markedly improved left ventricular performance compared with untreated IR rats, as reflected by a significant reduction in LVEDP from 28.50 ± 4.23 mmHg in the IR group to 19.50 ± 4.37 mmHg in the Sin+Iri group (absolute decrease of 9.00 mmHg; *P* = 0.0128) and a concurrent increase in LVDP from 49.83 ± 6.17 mmHg in the IR group to 68.67 ± 8.18 mmHg in the Sin+Iri group (absolute increase of 18.84 mmHg; *P* = 0.0001) (Figure 3b,c). Similarly, myocardial contractility and relaxation indices were significantly improved, with +d*P*/d*t* rising from 1386 ± 79.06 to 1780 ± 82.83 mmHg/s (absolute increase of 394 mmHg/s; *P* < 0.0001) and –d*P*/d*t* increasing from 631.50 ± 57.00 to 858.20 ± 49.13 mmHg/s (absolute increase of 226.70 mmHg/s; *P* = 0.0003) (Figure 3e,f). This combination therapy also demonstrated superior efficacy over individual pre‐treatments, with significantly greater improvements in LVDP (*P* = 0.0283 vs. sinomenine; *P* = 0.0253 vs. irisin), +d*P*/d*t* (*P* = 0.0007 vs. sinomenine; *P* = 0.0017 vs. irisin), and –d*P*/d*t* (*P* = 0.0316 vs. sinomenine; *P* = 0.0149 vs. irisin) (Figure 3c,e,f).

### 3.4 Effects of sinomenine and irisin on pyroptosis and mitophagy‐related proteins

**FIGURE 4:**
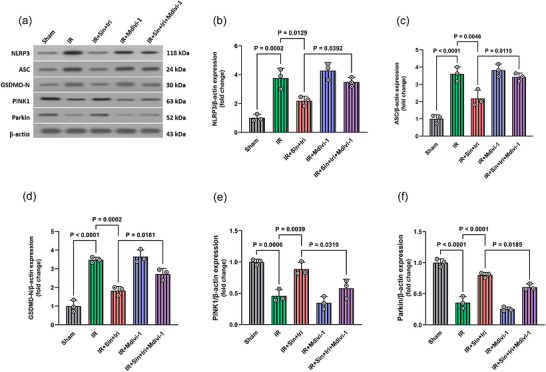
Proteins related to pyroptosis and mitophagy across experimental groups. Representative western blot bands (a) and protein expression levels of nucleotide‐binding oligomerization domain‐like receptor protein 3 (NLRP3) (b), apoptosis‐associated speck‐like protein containing a CARD (ASC) (c) and gasdermin D N‐terminal (GSDMD‐N) (d) as pyroptosis‐related proteins; and PTEN‐induced putative kinase 1 (PINK1) (e) and Parkin (f) as mitophagy‐related proteins. Data are expressed as means ± standard deviation (*n* = 3 per group) and statistically analysed using one‐way ANOVA with Tukey's *post hoc* test. IR, ischaemia–reperfusion; Iri, irisin; Mdivi‐1, mitochondrial division inhibitor 1; Sin, sinomenine.

Since the first protocol showed enhanced cardioprotective effects of the combined therapy compared to single‐agent pre‐treatments, we investigated the mechanisms responsible for this synergy. In this experimental setting, a mitophagy inhibitor (Mdivi‐1) was administered exclusively to the combination therapy group to further investigate its effect on cardioprotection. The protein expression levels of NLRP3, ASC, GSDMD‐N, PINK1 and Parkin were determined by western blot analysis, as illustrated in Figure 4a. The results showed that compared to the Sham group, the IR group had significantly higher expression of NLRP3 (*P* = 0.0002), ASC (*P* < 0.0001) and GSDMD‐N (*P* < 0.0001) and lower expression of PINK1 (*P* = 0.0006) and Parkin (*P* < 0.0001) (Figure 4b–f). In contrast, hearts from rats pre‐treated with the combination therapy had significantly lower NLRP3 (*P* = 0.0129), ASC (*P* = 0.0046) and GSDMD‐N (*P* = 0.0002) and higher PINK1 (*P* = 0.0039) and Parkin (*P* < 0.0001) compared to the IR group. Moreover, Mdivi‐1 administration together with sinomenine and irisin significantly reversed these trends, as evidenced by higher levels of NLRP3 (*P* = 0.0392), ASC (*P* = 0.0115) and GSDMD‐N (*P* < 0.0181) and lower PINK1 (*P* = 0.0319) and Parkin (*P* = 0.0185) compared to rats pre‐treated only with the combination therapy (Figure 4b–f).

### 3.5 Effects of sinomenine and irisin on pro‐inflammatory cytokine levels

**FIGURE 5:**
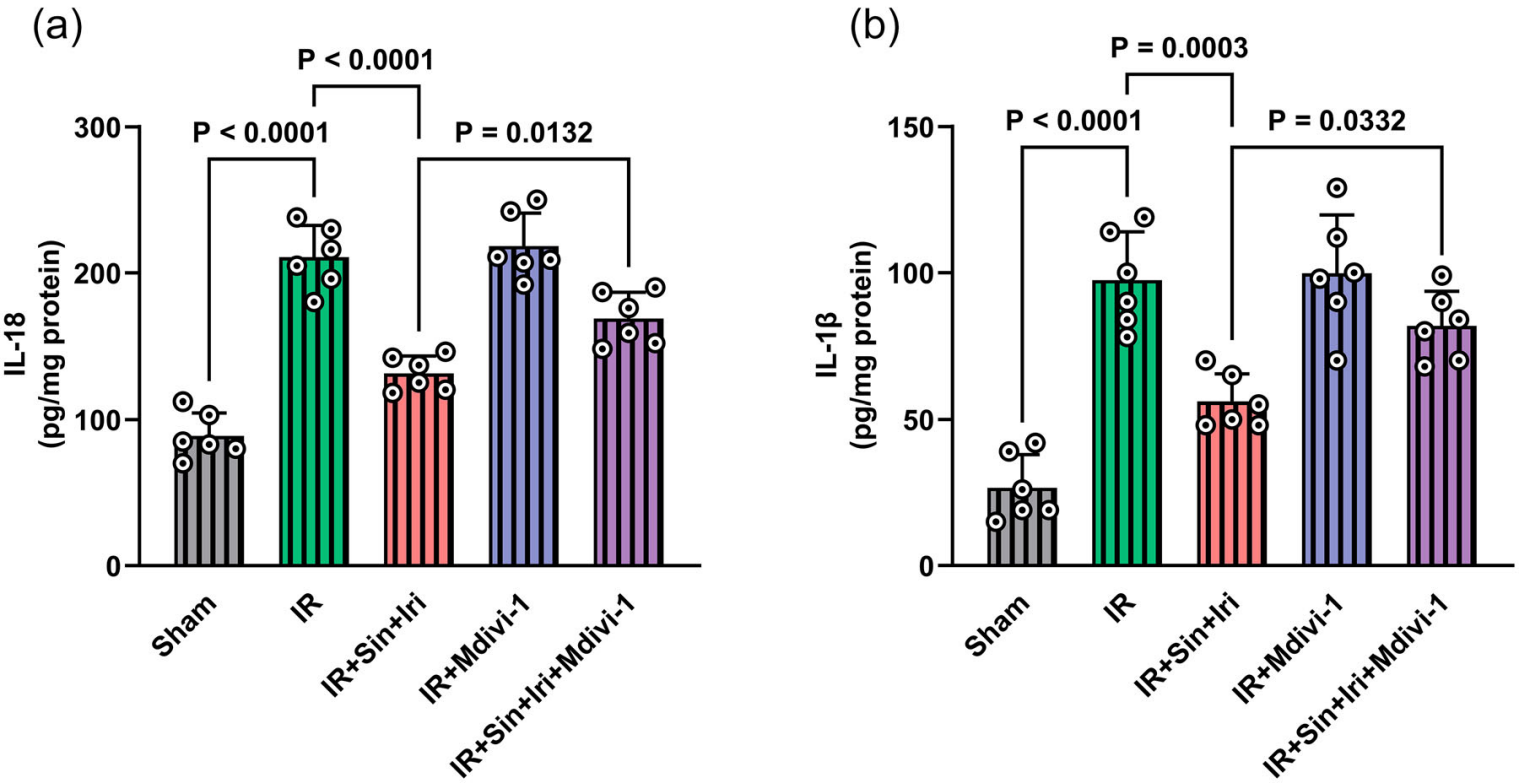
Levels of pro‐inflammatory cytokines across experimental groups. Interleukin‐18 (IL‐18) (a) and interleukin‐1β (IL‐1β) (b). Data are expressed as means ± standard deviation (*n* = 6 per group) and statistically analysed using one‐way ANOVA with Tukey's *post hoc* test. IR, ischaemia–reperfusion; Iri, irisin; Mdivi‐1, mitochondrial division inhibitor 1; Sin, sinomenine.

Our data showed that the levels of IL‐18 and IL‐1β were significantly higher in the IR group compared to the Sham group, and the difference in both was statistically significant (*P* < 0.0001) (Figure 5a,b). Combination pre‐treatment significantly blunted such elevations and resulted in lower concentrations of IL‐18 and IL‐1β in IR hearts (*P* < 0.0001 and *P* = 0.0003, respectively) (Figure 5a,b). Co‐administration of Mdivi‐1 with sinomenine and irisin displayed a less significant reduction in IL‐18 and IL‐1β levels compared to the combination therapy group (*P* = 0.0132 and *P* = 0.0332, respectively) (Figure 5a,b).

### 3.6 Effects of sinomenine and irisin on oxidative stress status

**FIGURE 6:**
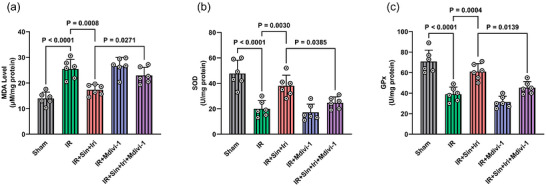
Oxidative stress status across experimental groups. The levels of malondialdehyde (MDA) (a) and the activities of superoxide dismutase (SOD) (b) and glutathione peroxidase (GPx) (c). Data are expressed as means ± standard deviation (*n* = 6 per group) and statistically analysed using one‐way ANOVA with Tukey's *post hoc* test. IR, ischaemia–reperfusion; Iri, irisin; Mdivi‐1, mitochondrial division inhibitor 1; Sin, sinomenine.

Data presented in Figure 6a–c show that oxidative stress was significantly enhanced in the IR group, characterized by higher MDA levels and concurrent decreased activities of antioxidant enzymes SOD and GPx compared to the Sham group (all *P* < 0.0001). Combination therapy markedly reduced the extent of oxidative damage, as demonstrated by a significant reduction in MDA concentration (*P* = 0.0008), with restoration of SOD and GPx enzymatic activities (*P* = 0.0030 and *P* = 0.0004, respectively) compared to the IR group. However, the addition of Mdivi‐1 to this combination partially reversed these protective effects, as evidenced by higher MDA levels (*P* = 0.0271) and reduction in SOD (*P* = 0.0385) and GPx (*P* = 0.0139) activities compared to those receiving combination therapy alone (Figure 6a–c).

## 4 DISCUSSION

The present study investigated whether the co‐administration of sinomenine and irisin could offer better protection against myocardial IR injury in a rat model. Our results showed that pre‐treatment with these agents significantly reduced myocardial infarct size, lowered serum levels of cTn‐I and abated histopathological injury to the heart tissue. Functional assessments further showed improved cardiac performance after combination pre‐treatment. At the mechanistic level, the cardioprotective effects were attributed to the suppression of NLRP3 inflammasome‐mediated pyroptosis and the consequent inflammatory response. Of note, the enhancement of the PINK1/Parkin‐dependent mitophagy pathway seemed to importantly contribute to the observed cardioprotection by removing damaged mitochondria and thus sustaining cellular integrity in the stressed myocardium. This underscores the important role that controlled mitophagy plays in mitochondrial quality control and protection of the heart against IR‐induced injury.

The protective effects of sinomenine in IR injury models have been extensively investigated. Accumulating evidence indicates that sinomenine confers myocardial protection primarily through its potent anti‐inflammatory and antioxidative properties (Xia et al., 2022). It attenuates oxidative stress by reducing ROS and MDA levels while enhancing endogenous antioxidant defences, including SOD and glutathione (Gao et al., 2024; Lu et al., 2022). In addition, sinomenine suppresses the production of pro‐inflammatory cytokines, thereby mitigating inflammatory responses in cardiac tissue (Chen et al., 2024). Collectively, these effects contribute to reduced infarct size, preservation of cardiac function and attenuation of cardiomyocyte apoptosis in experimental IR models (Zhang et al., 2021). Although most of this evidence derives from cardiac IR studies, findings from cerebral IR models further support the ability of sinomenine to protect ischaemic tissues through modulation of oxidative stress and inflammation (Qiu et al., 2016; Yang et al., 2016). In parallel with the anti‐inflammatory and antioxidative properties of sinomenine, irisin has recently attracted attention as another promising cardioprotective agent in IR injury. Emerging studies demonstrate that irisin reduces myocardial infarct size and improves cardiac function by promoting mitochondrial biogenesis and preserving mitochondrial integrity. Moreover, irisin exerts significant anti‐inflammatory effects by inhibiting inflammasome activation and decreasing pro‐inflammatory cytokine levels (Liao et al., 2019; Wang et al., 2018; Xin et al., 2020). Beyond the myocardium, investigations in cerebral ischaemia models have revealed neuroprotective properties of irisin, including regulation of mitochondrial dynamics and attenuation of oxidative damage, further underscoring its systemic protective potential in IR injury (Asadi et al., 2018; Liu et al., 2020). Against this background, the present study is among the first to evaluate the combined administration of sinomenine and irisin in cardiac IR injury. Our findings demonstrate that combination therapy provides superior cardioprotection compared with either monotherapy. This enhanced efficacy is likely attributable to complementary and potentially synergistic mechanisms, whereby the robust anti‐inflammatory and antioxidative actions of sinomenine are integrated with the mitochondrial‐protective and metabolic regulatory effects of irisin, collectively amplifying myocardial protection.

To further delineate the molecular basis underlying the superior cardioprotective efficacy of combined sinomenine and irisin therapy, we evaluated its effects on pyroptosis, mitophagy and oxidative stress – three interconnected processes critically involved in myocardial IR injury. Our findings demonstrate that the combination therapy more effectively suppressed pyroptotic signalling and its downstream inflammatory cascade than either monotherapy. Concurrently, it markedly enhanced PINK1/Parkin‐dependent mitophagy and reduced oxidative stress, suggesting coordinated regulation of mitochondrial quality control and inflammatory cell death pathways. The central contribution of mitophagy was substantiated using the selective mitophagy inhibitor Mdivi‐1. Pharmacological inhibition significantly attenuated – but did not entirely abolish – the cardioprotective effects of the combined pre‐treatment, indicating that PINK1/Parkin‐mediated mitophagy represents a major, but not exclusive, mechanistic axis. Mechanistically, activation of this pathway facilitates the selective clearance of dysfunctional mitochondria, preserves mitochondrial integrity, restrains excessive mitochondrial ROS generation, and consequently prevents inflammasome activation that drives pyroptosis and inflammatory amplification (Ji et al., 2022). Importantly, the superior efficacy of the combined regimen likely stems from the simultaneous modulation of multiple convergent protective pathways. Sinomenine predominantly suppresses inflammatory signalling cascades, including NF‐κB activation and downstream cytokine production (Xia et al., 2022), whereas irisin promotes mitochondrial biogenesis and metabolic reprogramming via AMP‐activated protein kinase (AMPK) signalling (Xin et al., 2020). The integration of these complementary mechanisms – anti‐inflammatory, antioxidative and mitochondrial‐regulatory – may produce additive or potentially synergistic cardioprotection, thereby achieving greater attenuation of oxidative damage, inflammatory responses and cardiomyocyte death compared with single‐agent therapy. Consistent with this concept, previous combination‐based cardioprotective strategies have demonstrated that concurrent targeting of multiple pathological pathways yields superior outcomes relative to monotherapies (Mokhtari & Badalzadeh, 2023). Collectively, these findings suggest that coordinated enhancement of mitophagy alongside complementary cytoprotective mechanisms constitutes a promising therapeutic approach for mitigating myocardial IR injury.

### 4.1 Limitations and future perspectives

Despite the robust cardioprotective effects observed with combined sinomenine and irisin therapy, several important limitations should be acknowledged to appropriately contextualize these findings within a translational framework. In line with the Improving Preclinical Assessment of Cardioprotective Therapies (IMPACT) criteria for early discovery‐phase studies, the present investigation represents a first‐phase evaluation conducted in young, healthy animals of a single sex, a design intentionally selected to maximize mechanistic resolution and reduce biological variability (Lecour et al., 2021). This approach was essential to precisely delineate the interplay between PINK1/Parkin‐dependent mitophagy and NLRP3‐mediated pyroptosis; however, it does not fully capture the cardioprotective resistance commonly observed in heterogeneous clinical populations. Accordingly, subsequent phases of investigation will expand this work to include both sexes as well as aged animals with clinically relevant comorbidities, particularly type 2 diabetes mellitus and hypertension, to determine whether the synergistic efficacy of sinomenine and irisin is maintained in hearts characterized by pre‐existing mitochondrial dysfunction and chronic inflammatory stress. Another limitation relates to the use of a pre‐treatment (pharmacological conditioning) paradigm. While this strategy is highly applicable to clinical settings involving anticipated ischaemia, such as elective coronary artery bypass grafting, percutaneous coronary interventions and cardiac transplantation, it does not directly address the unpredictable nature of spontaneous acute myocardial infarction. Therefore, future studies should evaluate the effectiveness of this combined therapy under post‐conditioning and rescue paradigms, with emphasis on long‐term functional recovery, adverse ventricular remodelling and survival outcomes. Finally, this study focused on PINK1/Parkin‐dependent mitophagy due to its key role in mitochondrial quality control during myocardial IR injury. Bcl‐2/adenovirus E1B 19 kDa‐interacting protein 3 (BNIP3)‐mediated, Parkin‐independent mitophagy is also relevant but was beyond the scope of this investigation and could be explored in future studies for additional mechanistic insight.

### 4.2 Conclusion

The present study demonstrates that the combined pre‐treatment with sinomenine and irisin confers superior cardioprotection against myocardial IR injury compared with either agent alone. This combinatorial strategy synergistically reduced myocardial infarct size, attenuated pyroptosis and its associated inflammatory responses, enhanced PINK1/Parkin‐dependent mitophagy and mitigated oxidative stress. By simultaneously targeting multiple convergent signalling pathways, the combination therapy effectively integrated the potent anti‐inflammatory and antioxidative properties of sinomenine with the mitochondrial‐protective and metabolic regulatory actions of irisin. This coordinated modulation of interconnected cellular processes resulted in a more comprehensive protective profile that exceeded the efficacy of monotherapy. Collectively, these findings provide a compelling rationale for multi‐target therapeutic strategies in the management of myocardial IR injury. Future investigations are warranted to further elucidate the molecular cross‐talk underlying this synergistic protection and to evaluate the translational potential of this combinational approach.

## AUTHOR CONTRIBUTIONS

Zhuzhu Liu coordinated the project and drafted the initial manuscript. Lianjun Wu and Sining Bi performed the animal experiments, collected data, and conducted the biochemical and molecular analyses. Amany Belal carried out the statistical analysis, contributed to data interpretation, and assisted in manuscript drafting and editing. Zhuzhu Liu conceived and designed the study, supervised the experimental procedures, and finalized the submitted version. All authors have read and approved the final version of this manuscript and agree to be accountable for all aspects of the work in ensuring that questions related to the accuracy or integrity of any part of the work are appropriately investigated and resolved. All persons designated as authors qualify for authorship, and all those who qualify for authorship are listed.

## CONFLICT OF INTEREST

The authors confirm that no financial, personal, or professional interests exist that might have biased the research or the preparation of this manuscript.

## FUNDING INFORMATION

None.

## Data Availability

The data that support the findings of this study are available from the corresponding author upon reasonable request.
